# Artificial intelligence in medical imaging: threat or opportunity? Radiologists again at the forefront of innovation in medicine

**DOI:** 10.1186/s41747-018-0061-6

**Published:** 2018-10-24

**Authors:** Filippo Pesapane, Marina Codari, Francesco Sardanelli

**Affiliations:** 1 Postgraduate School in Radiodiagnostics, Università degli Studi di Milano, Via Festa del Perdono 7, 20122 Milan, Italy; 2 Unit of Radiology, IRCCS Policlinico San Donato, Via Morandi 30, 20097 San Donato Milanese, Milan, Italy; 3 Department of Biomedical Sciences for Health, Università degli Studi di Milano, Via Morandi 30, 20097 San Donato Milanese, Milan, Italy

**Keywords:** Neural networks (computer), Artificial intelligence, Deep learning, Machine learning, Radiology

## Abstract

One of the most promising areas of health innovation is the application of artificial intelligence (AI), primarily in medical imaging. This article provides basic definitions of terms such as “machine/deep learning” and analyses the integration of AI into radiology. Publications on AI have drastically increased from about 100–150 per year in 2007–2008 to 700–800 per year in 2016–2017. Magnetic resonance imaging and computed tomography collectively account for more than 50% of current articles. Neuroradiology appears in about one-third of the papers, followed by musculoskeletal, cardiovascular, breast, urogenital, lung/thorax, and abdomen, each representing 6–9% of articles. With an irreversible increase in the amount of data and the possibility to use AI to identify findings either detectable or not by the human eye, radiology is now moving from a subjective perceptual skill to a more objective science. Radiologists, who were on the forefront of the digital era in medicine, can guide the introduction of AI into healthcare*.* Yet, they will not be replaced because radiology includes communication of diagnosis, consideration of patient’s values and preferences, medical judgment, quality assurance, education, policy-making, and interventional procedures. The higher efficiency provided by AI will allow radiologists to perform more value-added tasks, becoming more visible to patients and playing a vital role in multidisciplinary clinical teams.

## Key points

- Over 10 years, publications on AI in radiology have increased from 100–150 per year to 700–800 per year
- Magnetic resonance imaging and computed tomography are the most involved techniques
- Neuroradiology appears as the most involved subspecialty (accounting for about one-third of the papers), followed by musculoskeletal, cardiovascular, breast, urogenital, lung/thorax, and abdominal radiology (each representing 6–9% of articles)
- Radiologists, the physicians who were on the forefront of the digital era in medicine, can now guide the introduction of AI in healthcare

## Introduction

One of the most promising areas of health innovation is the application of artificial intelligence (AI) in medical imaging, including, but not limited to, image processing and interpretation [1]. Indeed, AI may find multiple applications, from image acquisition and processing to aided reporting, follow-up planning, data storage, data mining, and many others. Due to this wide range of applications, AI is expected to massively impact the radiologist’s daily life.

This article provides basic definitions of terms commonly used when discussing AI applications, analyses various aspects related to the integration of AI in the radiological workflow, and provides an overview of the balance between AI threats and opportunities for radiologists. Awareness of this trend is a necessity, especially for younger generations who will face this revolution.

## Artificial intelligence: definitions

**Fig. 1:**
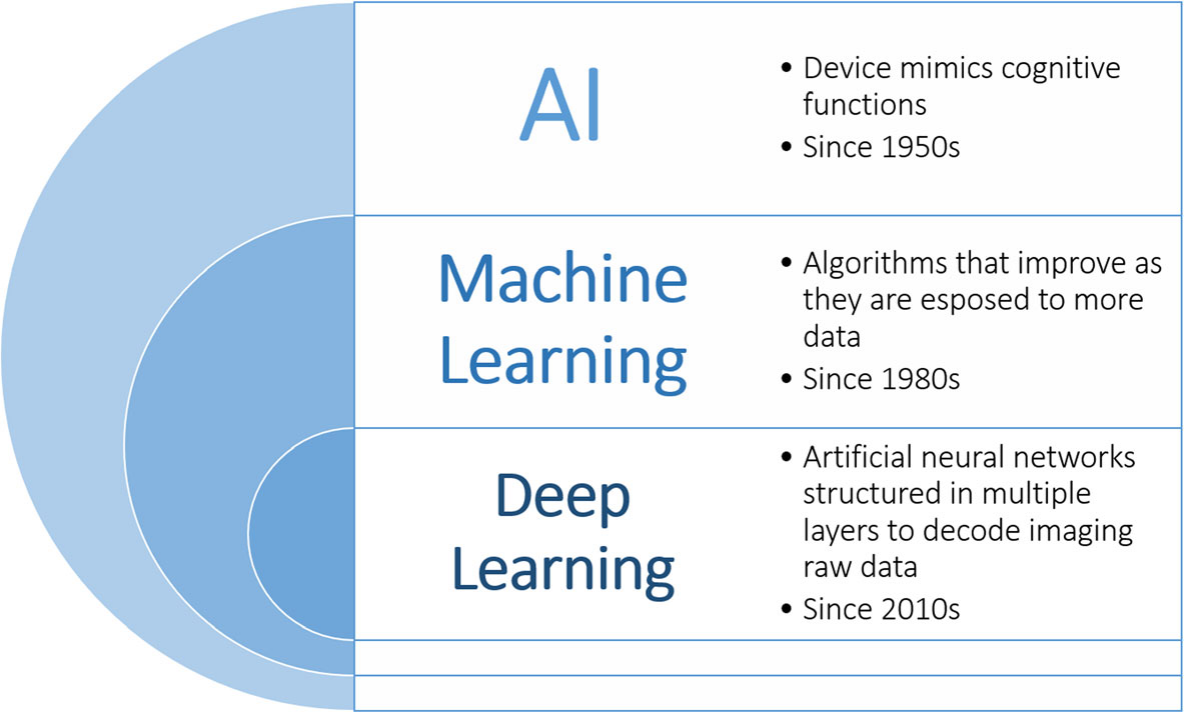
Deep learning as a subset of machine learning methods, which represent a branch of the existing artificial intelligence techniques. Machine learning techniques have been extensively applied since the 1980s. Deep learning has been applied since the 2010s due to the advancement of computational resources

The term AI is applied when a device mimics cognitive functions, such as learning and problem solving [2]. More generally, AI refers to a field of computer science dedicated to the creation of systems performing tasks that usually require human intelligence, branching off into different techniques [3]. *Machine learning* (ML), a term introduced by Arthur Samuel in 1959 to describe a subfield of AI [4] that includes all those approaches that allow computers to learn from data without being explicitly programmed, has been extensively applied to medical imaging [5]. Among the techniques that fall under the ML umbrella, *deep learning* (DL) has emerged as one of the most promising. Indeed, DL is a technique belonging to ML, which in turn refers to a broader AI family (Fig. 1). In particular, DL methods belong to representation-learning methods with multiple levels of representation, which process raw data to perform classification or detection tasks [6].

**Fig. 2:**
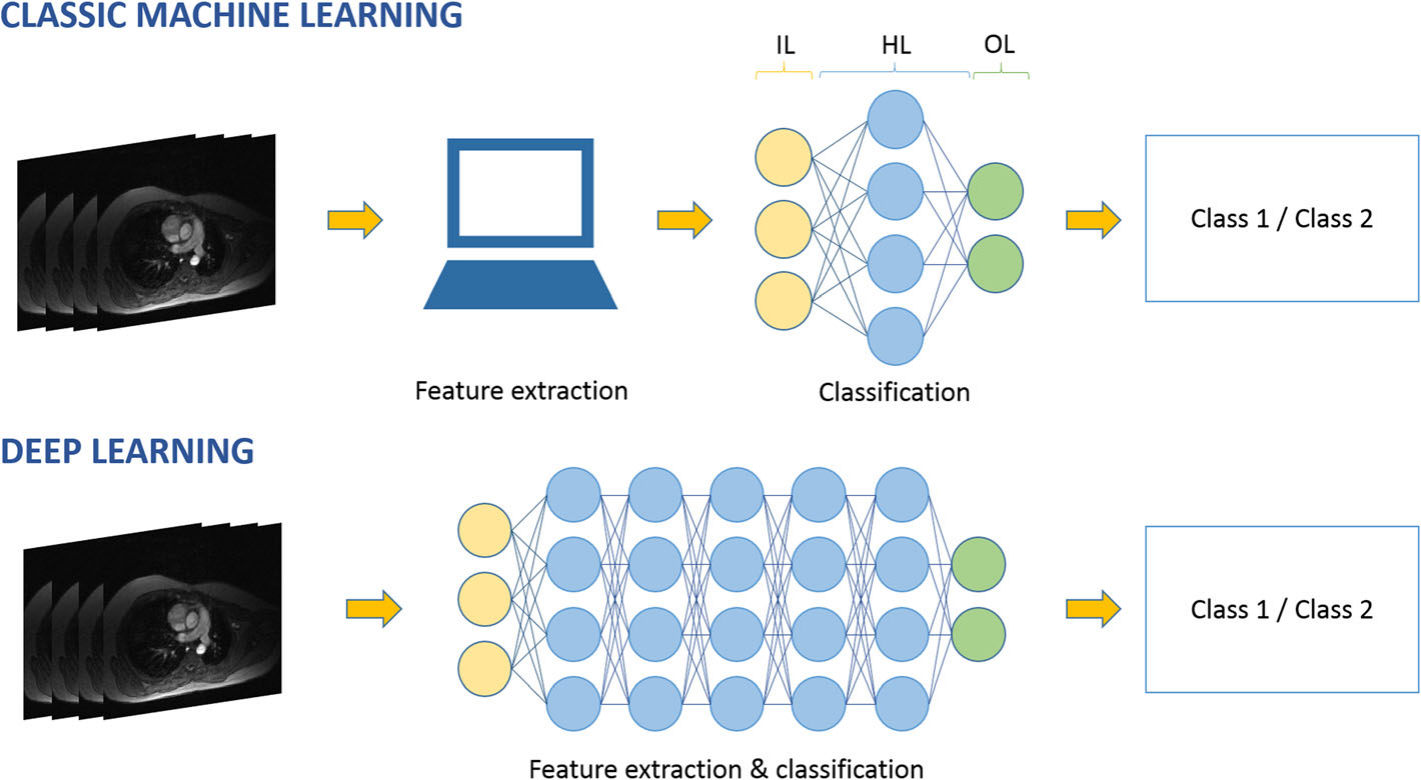
Comparison between classic machine learning and deep learning approaches applied to a classification task. Both depicted approaches use an artificial neural network organised in different layers (*IL* input layer, *HL* hidden layer, *OL* output layer). The deep learning approach avoids the design of dedicated feature extractors by using a deep neural network that represents complex features as a composition of simpler ones

ML incorporates computational models and algorithms that imitate the architecture of the biological neural networks in the brain, *i.e.*, *artificial neural networks* (ANNs) [7]. Neural network architecture is structured in layers composed of interconnected nodes. Each node of the network performs a weighted sum of the input data that are subsequently passed to an activation function. Weights are dynamically optimised during the training phase. There are three different kinds of layers: the input layer, which receives input data; the output layer, which produces the results of data processing; and the hidden layer(s), which extracts the patterns within the data. The DL approach was developed to improve on the performance of conventional ANN when using deep architectures. A *deep* ANN differs from the single hidden layer by having a large number of hidden layers, which characterise the depth of the network [8]. Among the different deep ANNs, *convolutional neural networks* (CNNs) have become popular in computer vision applications. In this class of deep ANNs, *convolution* operations are used to obtain feature maps in which the intensities of each pixel/voxel are calculated as the sum of each pixel/voxel of the original image and its neighbours, weighted by convolution matrices (also called *kernels*). Different kernels are applied for specific tasks, such as blurring, sharpening, or edge detection. CNNs are biologically inspired networks mimicking the behaviour of the brain cortex, which contains a complex structure of cells sensitive to small regions of the visual field [3]. The architecture of deep CNNs allows for the composition of complex features (such as shapes) from simpler features (e.g. image intensities) to decode image raw data without the need to detect specific features [3] (Fig. 2).

Despite their performance, ML network architecture makes them more prone to fail in reaching the convergence and overfit training dataset. On the other hand, the complexity of deep network architectures makes them demanding in terms of computational resources and dimension of the training sample.

**Fig. 3:**
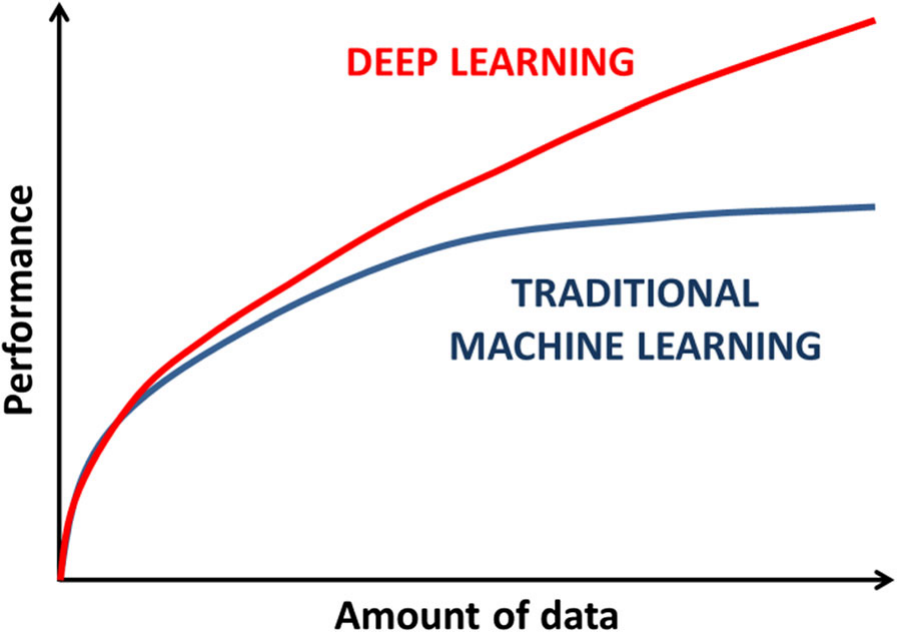
Graphical representation of the different relationship between the amount of data given to traditional ML or DL systems and their performance. Only DL systems continue to increase their performance

Success in DL application was possible mainly due to recent advancements in the development of hardware technologies, like graphics processing units [5]. Indeed, the high number of nodes needed to detect complex relationships and patterns within data may result in billions of parameters that need to be optimised during the training phase. For this reason, DL networks require a huge amount of training data, which in turn increase the computing power needed to analyse them. These are also the reasons why DL algorithms are showing increased performance and are, theoretically, not susceptible to the performance plateau of the simpler ML networks (Fig. 3).

**Fig. 4:**
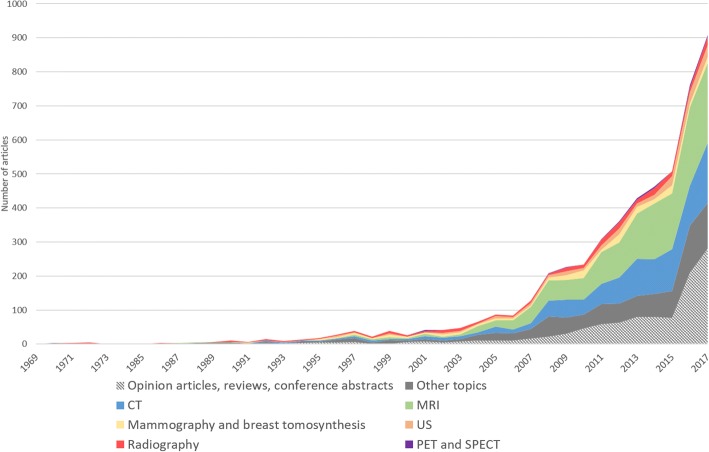
Number of publications indexed on EMBASE obtained using the search query (‘artificial intelligence’/exp. OR ‘artificial intelligence’ OR ‘machine learning’/exp. OR ‘machine learning’ OR ‘deep learning’/exp. OR ‘deep learning’) AND (‘radiology’/exp. OR ‘radiology’ OR ‘diagnostic imaging’/exp. OR ‘diagnostic imaging’) AND ([english]/lim). EMBASE was accessed on April 24, 2018. For each year the number of publications was stratified for imaging modality. *US* ultrasound, *MRI* magnetic resonance imaging, *CT* computed tomography, *PET* positron emission tomography, *SPECT* single-photon emission tomography. Diagnostic modalities different from those listed above are grouped under the “other topic” label (e.g. optical coherence tomography, dual-energy x-ray absorptiometry, etc.)

Radiologists are already familiar with computer-aided detection/diagnosis (CAD) systems, which were first introduced in the 1960s in chest x-ray and mammography applications [5]. However, advances in algorithm development, combined with the ease of access to computational resources, allows AI to be applied in radiological decision-making at a higher functional level [7].

## The wind of change

**Fig. 5:**
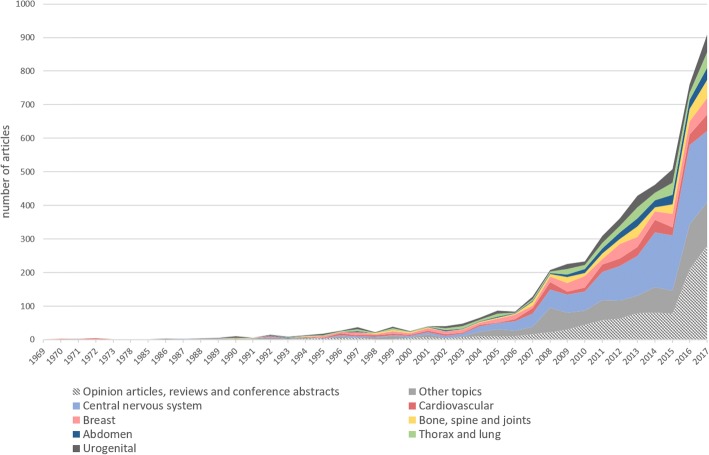
Number of publications indexed on EMBASE obtained using the search query (‘artificial intelligence’/exp. OR ‘artificial intelligence’ OR ‘machine learning’/exp. OR ‘machine learning’ OR ‘deep learning’/exp. OR ‘deep learning’) AND (‘radiology’ OR ‘diagnostic imaging’). EMBASE was accessed on April 24, 2018. For each year, the number of publications was subdivided separating opinion articles, reviews and conference abstracts from original articles in seven main subgroups considering subspecialty or body part. Other fields of medical imaging different from those listed above are grouped under the “other topics” label (e.g. dermatology, ophthalmology, head and neck, etc.)

The great enthusiasm for and dynamism in the development of AI systems in radiology is shown by the increase in publications on this topic (Figs. 4 and 5). Only 10 years ago, the total number of publications on AI in radiology only just exceeded 100 per year. Thereafter, we had a tremendous increase, with over 700–800 publications per year in 2016–17. In the last couple of years, computed tomography (CT) and magnetic resonance imaging (MRI) have collectively accounted for more than 50% of articles, though radiography, mammography, and ultrasound are also represented (Table 1). Neuroradiology (here evaluated as imaging of the central nervous system) is the most involved subspecialty (accounting for about one-third of the papers), followed by musculoskeletal, cardiovascular, breast, urogenital, lung/thorax, and abdominal radiology, each representing between 6 and 9% of the total number of papers (Table 2). AI currently has an impact on the field of radiology, with MRI and neuroradiology as the major fields of innovation.

**Table 1:** Number of articles on AI in radiology indexed on EMBASE, stratified by imaging modality

Imaging modality	2015	2016	2017
Magnetic resonance imaging	164	230	235
	38%	42%	37%
Computed tomography	123	117	177
	29%	21%	28%
Ultrasound	27	32	33
	6%	6%	5%
Radiography	14	14	26
	3%	3%	4%
Mammography and breast tomosynthesis	23	12	18
	5%	2%	3%
Positron emission tomography and single-photon emission tomography	1	7	5
	0%	1%	1%
Other	79	139	134
	18%	25%	21%
Total	431	551	628
	100%	100%	100%

Search query: (‘artificial intelligence’/exp. OR ‘artificial intelligence’ OR ‘machine learning’/exp. OR ‘machine learning’ OR ‘deep learning’/exp. OR ‘deep learning’) AND (‘radiology’/exp. OR ‘radiology’ OR ‘diagnostic imaging’/exp. OR ‘diagnostic imaging’) AND ([english]/lim). Values were obtained including only “article”, “article in press” and “conference paper” as publication type. EMBASE was accessed on April 24, 2018

**Table 2:** Number of articles indexed on EMBASE stratified by radiology subspecialty/body part

Body parts	2015	2016	2017
Central nervous system	163	235	211
	38%	43%	34%
Bone, spine and joints	29	37	54
	7%	7%	9%
Cardiovascular	24	32	49
	6%	6%	8%
Breast	41	39	50
	10%	7%	8%
Urogenital	40	25	52
	9%	5%	8%
Thorax and lungs	36	21	46
	8%	4%	7%
Abdomen	28	27	36
	6%	5%	6%
Other	70	135	130
	16%	25%	21%
Total	431	551	628
	100%	100%	100%

Search query: (‘artificial intelligence’/exp. OR ‘artificial intelligence’ OR ‘machine learning’/exp. OR ‘machine learning’ OR ‘deep learning’/exp. OR ‘deep learning’) AND (‘radiology’/exp. OR ‘radiology’ OR ‘diagnostic imaging’/exp. OR ‘diagnostic imaging’) AND ([english]/lim) stratified by radiology subspeciality/body parts. Values were obtained including only “article”, “article in press” and “conference paper” as publication type. EMBASE was accessed on April 24, 2018

Recent meetings have also proven the interest in AI applications. During the 2018 European Congress of Radiology and the 2017 Annual Meeting of the Radiological Society of North America, AI represented the focus of many talks. Studies showed the application of DL algorithms for assessing the risk of malignancy for a lung nodule, estimating skeletal maturity from paediatric hand radiographs, classifying liver masses, and even obviating the need for thyroid and breast biopsies [9–11]; at the same time, the vendors showed examples of AI applications in action [9, 10].

## AI in radiology: threat or opportunity?

A motto of radiology residents is: “The more images you see, the more examinations you report, the better you get”. The same principle works for ML, and in particular for DL. In the past decades, medical imaging has evolved from projection images, such as radiographs or planar scintigrams, to tomographic (i.e. cross-sectional) images, such as ultrasound (US), CT, tomosynthesis, positron emission tomography, MRI, etc., becoming more complex and data rich. Even though this shift to three-dimensional (3D) imaging began during the 1930s, it was not until the digital era that this approach allowed high anatomic detail to be obtained and functional information to be captured.

The increasing amount of data to be processed can influence how radiologists interpret images: from inference to merely detection and description. When too much time is taken for image analysis, the time for evaluating clinical and laboratory contexts is squeezed [12]. The radiologist is reduced to being only an image analyst. The clinical interpretation of the findings is left to other physicians. This is dangerous, not only for radiologists but also for patients: non-radiologists can have a full understanding of the clinical situation but do not have the radiological knowledge. In other words, if radiologists do not have the time for clinical judgement, the final meaning of radiological examinations will be left to non-experts in medical imaging.

In this scenario, AI is not a threat to radiology. It is indeed a tremendous opportunity for its improvement. In fact, similar to our *natural intelligence*, AI algorithms look at medical images to identify patterns after being trained using vast numbers of examinations and images. Those systems will be able to give information about the characterisation of abnormal findings, mostly in terms of conditional probabilities to be applied to Bayesian decision-making [13, 14].

This is crucial because not all abnormalities are representative of disease and must be actioned. AI systems learn on a case-by-case basis. However, unlike CAD systems, which just highlight the presence or absence of image features known to be associated with a disease state [15, 16], AI systems look at specific labelled structures and also learn how to extract image features either visible or invisible to the human eye. This approach mimics human analytical cognition, allowing for better performance than that obtained with old CAD software [17].

With the irreversible increase in imaging data and the possibility to identify findings that humans can or cannot detect [18], radiology is now moving from a subjective perceptual skill to a more objective science [12]. In fact, the radiologist’s work is currently limited by subjectivity, i.e. variations across interpreters, and the adverse effect of fatigue. The attention to inter- and intra-reader variability [19] and the work committed to improve the repeatability and reproducibility of medical imaging over the past decades proves the need for reproducible radiological results. In a broader perspective, the trend toward data sharing also works in this case [20]. The key point is that AI has the potential to replace many of the routine detection, characterisation and quantification tasks currently performed by radiologists using cognitive ability, as well as to accomplish the integration of data mining of electronic medical records in the process [1, 7, 21].

Moreover, the recently developed DL networks have led to more robust models for radiomics, which is an emerging field that deals with the high-throughput extraction of quantitative peculiar features from radiological images [22–26]. Indeed, data derived from radiomics investigation, such as intensity, shape, texture, wavelength, etc., can be extracted from medical images [23, 27–31] and extracted by or integrated in ML approaches, providing valuable information for the prediction of treatment response, differentiating benign and malignant tumours, and assessing cancer genetics in many cancer types [23, 32–34]. Because of the rapid growth of this area, numerous published radiomics investigations lack standardised evaluation of both the scientific integrity and the clinical relevance [22]. However, despite the ongoing need for independent validation datasets to confirm the diagnostic and prognostic value of radiomics features, radiomics has shown several promising applications for personalised therapy [22, 23], not only in oncology but also in other fields, as shown by recent original articles that have proved the value of radiomics in the cardiovascular CT domain [35, 36].

Finally, AI applications may enhance the reproducibility of technical protocols, improving image quality and decreasing radiation dose, decreasing MRI scanner time [39] and optimising staffing and CT/MRI scanner utilisation, thereby reducing costs [1]. These applications will simplify and accelerate technicians’ work, also resulting in an average higher technical quality of examinations. This may counteract one of the current limitations of AI systems, i.e. the low ability to recognise the effects of positioning, motion artefacts, etc., also due to the lack of standardised acquisition protocols [15, 33, 34]. In other words, AI needs high-quality studies, but its application will lead towards better quality. The holy grail of standardisation in radiology may become attainable, also increasing productivity*.*

The quicker and standardised detection of image findings has the potential to shorten reporting time and to create automated sections of reports [2]. Structured AI-aided reporting represents a domain where AI may have a great impact, helping radiologists use relevant data for diagnosis and presenting it in a concise format [40].

Recently, the radiological community has discussed how such changes will alter the professional status of radiologists. Negative feelings were expressed, reflecting the opinions of those who are thinking that medicine will not need radiologists at all [21, 41, 42]. Should we consider closing postgraduate schools of radiology, as someone suggested [12, 21]? No. Healthcare systems would not be able to work without radiologists, particularly in the AI era. This answer is not based on a prejudicial defence of radiology as a discipline and profession. Two main ideas should guide this prediction: first, “The best qualification of a prophet is to have a good memory” (attributed to Marquis of Halifax) [43]; and second, “One way to predict the future is to create it” (attributed to Abraham Lincoln) [44].

Radiologists were on the forefront of the digital era in medicine. They guided the process, being the first medical professionals to adopt computer science, and are now probably the most digitally informed healthcare professionals [45]. Although the introduction of new technologies was mostly perceived as new approaches for producing images, innovation also deeply changed the ways to treat, present and store images. Indeed, the role of radiologists was strengthened by the introduction of new technologies. Why should it be different now? The lesson of the past is that apparently disrupting technologies (e.g. non-x-ray-based modalities, such as ultrasound and MRI) that seemed to go beyond radiology were embraced by radiologists. Radiology extended its meaning to radiation-free imaging modalities, now encompassing almost all diagnostic medical imaging, as demonstrated by the presence of the word “radiology” in numerous journals titles. This historical effect resulted from the capacity of radiologists to embrace these radiation-free modalities. In addition, electronic systems for reporting examination and archiving images were primarily modelled to serve radiologists.

The reasonable doubt is that we are now facing methods that not only cover the production of medical images but also involve detection and characterisation, properly entering the diagnostic process. Indeed, this is a new challenge, but also an additional value of AI. The professional role and satisfaction of radiologists will be enhanced by AI if they, as in the past, embrace this technology and educate new generations to use it to save time spent on routine and monotonous tasks, with strong encouragement to dedicate the saved time to functions that add value and influence patient care. This could also help radiologists feel less worried about the high number of examinations to be reported and rather focus on communication with patients and interaction with colleagues in multidisciplinary teams [46]. This is the way for radiologists to build their bright future.

We are at the beginning of the AI era. Until now, the clinical application of ML on medical imaging in terms of detection and characterisation have produced results limited to specific tasks, such as differentiation of normal from abnormal chest radiographs [41, 42, 47] or mammograms [48, 49]. The application of AI to advanced imaging modalities, such as CT and MRI, is now in its first phase. Examples of promising results are the differentiation of malignant from benign chest nodules on CT scans [50], the diagnosis of neurologic and psychiatric diseases [51, 52], and the identification of biomarkers in glioblastoma [53]. Interestingly, MRI has been shown to predict survival in women with cervical cancer [54, 55] and in patients with amyotrophic lateral sclerosis [56].

However, AI could already be used to accomplish tasks with a positive, immediate impact, several of them already described by Nance et al. in 2013 [57]:

1. *Prioritisation of reporting*: automatic selection of findings deserving faster action.
2. *Comparison of current and previous examinations, especially in oncologic follow-up*: tens of minutes are needed for this currently; AI could do this for us; we will supervise the process, extracting data to be integrated into the report and drawing conclusions considering the clinical context and therapy regimens; AI could also take into account the time interval between examinations
3. *Quick identification of negative studies*: at least in this first phase, AI will favour sensitivity and negative predictive value over specificity and positive predictive value, finding the normal studies and leaving abnormal ones for radiologists [36]. This would be particularly useful in high-volume screening settings; this concept of *quick negative* should also represent a helpful tool for screening programmes in underserved countries [10, 37].
4. *Aggregation of electronic medical records*, allowing radiologists to access clinical information to adapt protocols or interpret exams in the full clinical context.
5. *Automatic recall and rescheduling of patients*: for findings deserving of an imaging follow-up.
6. *Immediate use of clinical decision support systems* for ordering, interpreting, and defining further patient management.
7. *Internal peer-review of reports.*
8. *Tracking of residents’ training*.
9. *Quality control of technologists’ performance and tracked communication between radiologists and technologists*.
10. *Data mining regarding relevant issues*, including radiation dose [38].

In the mid-term perspective, other possibilities are open, such as:

1. *Anticipation of the diagnosis of cancerous lesions in oncologic patients* using texture analysis and other advanced approaches [58].
2. *Prediction of treatment response to therapies for tumours*, such as intra-arterial treatment for hepatocellular carcinoma [59].
3. *Evaluation of the biological relevance of borderline cases*, such as B3-lesions diagnosed at pathology of needle biopsy of breast imaging findings [60].
4. *Estimation of functional parameters*, such as the fractional flow reserve from CT coronary angiography using deep learning [61].
5. *Detection of perfusion defects and ischaemia*, for example in the case of myocardial stress perfusion defects and induced ischaemia [62].
6. *Segmentation and shape modelling*, such as brain tumour segmentation [63] or, more generally, brain structure segmentation [64].
7. *Reducing diffusion MRI data processing to a single optimised step*, for example making microstructure prediction on a voxel-by-voxel basis as well as automated model-free segmentation from MRI values [39].

## The radiologists’ role and cooperation with computer scientists

The key point is the separation of diagnosis and prediction from action and recommendation. Radiologists will not be replaced by machines because radiology practice is much more than the simple interpretation of images*.* The radiologist’s duties also include communication of diagnosis, consideration of patients’ values and preferences, medical judgment, quality assurance, education, policy-making, interventional procedures, and many other tasks that, so far, cannot be performed by computer programmes alone [2].

Notably, it must be understood that the clinical role of radiologists cannot be “saved” only by performing interventional procedures. Even though interventional radiology is a fundamental asset to improve the clinical profile of radiology [45, 65–67], radiologists must act more as clinicians, applying their clinical knowledge in answering diagnostic questions and guiding decision-making, which represent their main tasks. Radiologists should keep their human control in the loop, considering clinical, personal and societal contexts in ways that AI alone is not able to do. So far, AI is neither astute nor empathic. Thus, physicians (i.e., we say here radiologists) remain essential for medical practice, because ingenuity in medicine requires unique human characteristics [40, 68]. If the time needed for image interpretation were shortened, radiologists would be able to focus on inference to improve patient care. If AI is based on a huge increase in information, the hallmark of intelligence is in reducing information to what is relevant [69].

However, it is impossible to exclude that the efficiency gain provided by AI may lead to a need for fewer radiologists. Of note, in the United States the competition for residency positions in radiology has decreased over time [70], potentially allowing for a better match between supply and demand for residency positions. However, also the opposite hypothesis cannot be excluded: AI-enhanced radiology may require more professionals in the field, including radiologists. In general, the history of automation shows that jobs are not lost. Rather, roles are reshaped; humans are displaced to tasks needing a human element [12]. The gain in efficiency provided by AI will allow radiologists to perform more value-added tasks, such as integrating patients’ clinical and imaging information, having more professional interactions, becoming more visible to patients and playing a vital role in integrated clinical teams [46]. In this way, AI will not replace radiologists; yet, those radiologists who take advantage of the potential of AI will replace the ones who refuse this crucial challenge.

Finally, we should remember that AI mimics human intelligence. Radiologists are key people for several current AI challenges, such as the creation of high-quality training datasets, definition of the clinical task to address, and interpretation of obtained results. Many labelled studies and findings provided by experienced radiologists are needed; those datasets are difficult to find and are time-consuming, implying high costs [3]. However, even if crucial, the radiologist’s role should not be confined to data labelling. Radiologists may play a pivotal role in the identification of clinical applications where AI methods may make the difference. Indeed, they represent the final user of these technologies, who knows where they can be applied to improve patient care. For this reason, their point of view is crucial to optimise the use of AI-based solutions in the clinical setting. Finally, the application of AI-based algorithms often leads to the creation of complex data that need to be interpreted and linked to their clinical utility. In this scenario, radiologists may play a crucial role in data interpretation, cooperating with data scientists in the definition of useful results.

Radiologists should negotiate the supply of these valuable datasets and clinical knowledge with a guiding role in the clinical application of AI programmes*.* This implies an increasing partnership with bioengineers and computer scientists. Working with them in research and development of AI applications in radiology is a strategic issue [45]. These professionals should be embedded in radiological departments, becoming everyday partners. Creating this kind of “multidisciplinary AI team” will help to ensure patient safety standards are met and creates judicial transparency, which allows legal liability to be assigned to the radiologist component human authority [71].

Another topical issue that needs to be faced are the legal implications of AI systems in healthcare. As soon as AI systems start making autonomous decisions about diagnoses and prognosis, and stop being only a support tool, a problem arises as to whether, when something ‘goes wrong’ following a clinical decision made by an AI application, the reader (namely, the radiologist) or the device itself or its designer/builder is to be considered at fault [72]. In our opinion, ethical and legal responsibility for decision making in healthcare will remain a matter of the natural intelligence of physicians. From this viewpoint, it is probable that the multidisciplinary AI team will take responsibility in difficult cases, considering relevant, but not always conclusive, what AI provided. It has been already demonstrated that groups of human and AI agents working together make more accurate predictions compared to humans or AI alone, promising to achieve higher levels of accuracy in imaging diagnosis and even prognosis [71].

Although the techniques of AI differ from diagnosis to prognosis, both applications still need validation, and this is challenging due to the large amount of data needed to achieve robust results [73]. Therefore, rigorous evaluation criteria and reporting guidelines for AI need to be developed in order to establish its role in radiology and, more generally, in medicine [22].

## Conclusions

AI will surely impact radiology, and more quickly than other medical fields. It will change radiology practice more than anything since Roentgen. Radiologists can play a leading role in this oncoming change [74].

An uneasiness among radiologists to embrace AI may be compared with the reluctance among pilots to embrace autopilot technology in the early days of automated aircraft aviation. However, radiologists are used to facing technological challenges because, since the beginnings of its history, radiology has been the playfield of technological development.

An updated radiologist should be aware of the basic principles of ML/DL systems, of the characteristic of datasets to train them, and their limitations. Radiologists do not need to know the deepest details of these systems, but they must learn the technical vocabulary used by data scientists to efficiently communicate with them. The time to work for and with AI in radiology is now.

## Abbreviations

3D: Three-dimensional
AI: Artificial intelligence
ANN: Artificial neural network
CAD: Computer-aided detection/diagnosis
CNN: Convolutional neural network
CT: Computed tomography
DL: Deep learning
ML: Machine learning
MRI: Magnetic resonance imaging
